# Exploration of Simiao-Yongan Decoction on knee osteoarthritis based on network pharmacology and molecular docking

**DOI:** 10.1097/MD.0000000000035193

**Published:** 2023-10-06

**Authors:** Ying Wang, Xiangyu Pan, Junwei Wang, Haixu Chen, Lan Chen

**Affiliations:** a Department of Basic Medicine, Sichuan Vocational College of Health and Rehabilitation, Zigong, Sichuan Province, China; b Department of Rehabilitation Medicine, Zigong First People’s Hospital, Zigong, Sichuan Province, China; c Department of Pediatric Surgery & Vascular Surgery, Zigong Fourth People’s Hospital, Zigong, Sichuan Province, China.

**Keywords:** apoptosis, knee osteoarthritis, Simiao-Yongan Decoction, TCM

## Abstract

Use network pharmacology combined with molecular docking to study the effects of Simiao-Yongan Decoction (SMYAD) intervenes in Knee Osteoarthritis (KOA) related targets and signaling pathways, and explores the molecular mechanism of SMYAD in treating KOA. The active ingredients and targets of SMYAD, which concluded 4 traditional Chinese medicines, were screened in TCMSP, and the related gene targets of KOA were screened in the disease databases GeneCards, MalaCards, DisGeNET, and Comparative Toxicogenomics Database, and their intersection data were obtained after integration. And used Cytoscape 3.9.1, the software topologies the network diagram of “compound—drug-active ingredient-target protein-disease.” Obtains the protein-protein interaction network diagram through STRING, and enriches and analyzes the obtained core targets. Carry out molecular docking matching verification on the main active ingredients and key targets of the drug. 106 active ingredients and 175 targets were screened from SMYAD to intervene in KOA, 36 core targets were obtained through protein-protein interaction screening, and 10 key targets played an important role. The enrichment results showed that the biological process of gene ontology mainly involved positive regulation of gene expression, negative regulation of apoptosis process, and positive regulation of apoptosis process. KEGG signaling pathway mainly involves AGE-RAGE signaling pathway in diabetic complications, TNF signaling pathway, hypoxia-inducible factor-1 signaling pathway, IL-17 signaling pathway. The pathway of Reactome mainly involves interleukin-4 and interleukin-13 signaling, cytokine signaling in immune system, immune system, apoptosis. Molecular docking showed that the mainly effective components of SMYAD can fully combine with TNF, IL1B, IL6, and CASP3. The results show that the main active ingredients and potential mechanism of action of SMYAD in the treatment of KOA have the characteristics of multiple targets and multiple pathways, which provides ideas and basis for further in-depth exploration of its specific mechanism.

## 1. Introduction

Knee osteoarthritis (KOA) is the most common joint inflammatory disease in orthopedics. The main feature is the gradual degeneration and loss of articular cartilage, accompanied by changes in joint structure and function.^[1]^ After entering the 21st century, because of the increase in life expectancy and increase in body mass index due to the increase of population of old people, the prevalence of KOA has increased significantly and tended to be younger.^[2]^ Currently, KOA accounts for 85% of the global burden of osteoarthritis, and the incidence of symptomatic KOA is 8.1%.^[3]^ The main clinical manifestations of KOA are inconvenient movement of the limbs, difficulty in moving, and pain and swelling around the joints, which are more obvious after waking up in the morning, and can lead to paralysis in severe cases in the later stage.^[2]^ Inflammation is the most common cause of joint pain and patient disability, and no effective treatment has been found to delay the progression of KOA, so most patients will undergo total joint replacement to relieve symptoms and improve physical performance, but it is expensive and traumatic large, slow recovery in the later period.^[4–6]^ Therefore, it is crucial to understand the pathogenic process of KOA and the cellular and molecular mechanisms involved. The current process of osteoarthritis of the knee contains 2 basic aspects: the action of different joint elements and posttraumatic osteoarthritis formation.^[7,8]^ On one hand, in nontraumatic circumstances, the development of knee osteoarthritis is a degenerative pathology associated with aging. Injuries to the components of the knee joint, such as synovitis, ligament, meniscus, free bodies, popliteal cysts, and chondromalacia of the patella, can also result in the development of knee osteoarthritis. In addition, knee osteoarthritis can be brought on by putting on too much weight, adopting bad gait patterns, squatting for extended periods of time, and exposing the knee joint to the cold.^[4,9]^ On the other hand, posttraumatic osteoarthritis development, a progressive form of arthritis in the knee following trauma, is a significant contributor to osteoarthritis of the knee. It has clinical symptoms with osteoarthritis, but it also has a clear history of trauma, such as transarticular fractures.^[10,11]^ At present, the most common manifestation of KOA is pain, and the effective drugs for pain relief are nonsteroidal antiinflammatory drugs.^[12]^ However, such drugs are only suitable for short-term pain relief, and cannot control the wear and tear of articular cartilage, and cannot solve the fundamental problem.^[13]^ Moreover, such drugs may cause gastrointestinal discomfort such as nausea and vomiting, and increase the risk of gastrointestinal bleeding.^[14]^ Therefore, it is imperative to develop a drug that can maintain the curative effect of the drug and reduce side effects as much as possible.

Traditional Chinese medicine (TCM) attributes KOA to the categories of “Bi Syndrome” and “Bone Bi.” TCM has a long history of treating KOA, and the basic theoretical system of “arthromyopathy” was established as early as in the “Huangdi Neijing.” TCM has the advantages of definite curative effect and high safety in the treatment of KOA. It has a good clinical effect on the treatment of KOA and plays a vital role in the diagnosis and treatment of KOA. Many doctors in modern times use Simiao-Yongan Decoction (SMYAD) to treat arthritis. The results of modern pharmacological research also show that SMYAD has antiinflammatory, analgesic, and microcirculation improvement functions,^[15]^ and there have been clinical research reports, SMYAD has a definite curative effect on gouty arthritis, but there is no relevant research on the active substances and specific molecular mechanisms of SMYAD.^[16]^

Network pharmacology is a new discipline formed by the fusion of pharmacology with multidisciplinary methods, which can comprehensively reflect the molecular mechanism of action of drugs on disease networks.^[17]^ In this study, using the methods of network pharmacology and molecular docking, starting from the chemical substance basis of SMYAD, the important targets and potential molecular mechanisms of drug regulation on KOA are more systematically and deeply explored, to better serve the treatment of KOA. It provides a theoretical reference for the clinical treatment of KOA and provides new ideas for the development of new drugs for the treatment of KOA.^[18]^

## 2. Materials and methods

### 2.1. Screen the active ingredients of SMYAD and the protein targets of KOA

Jin Yinhua (or honeysuckle), Xuan Shen (or Genuine Ginseng), Dang Gui (or angelica), and Gan Cao (or licorice) were collected through TCMSP (http://tcmspw.com/tcmsp.php) database. Taking the oral bioavailability ≥30% and drug similarity (DL) ≥0.18 as the standard, the effective active ingredients of the drug were screened, and the targets corresponding to the active ingredients were queried,^[19]^ targets to obtain effective targets. In GeneCards (https://www.genecards.org/), MalaCards (https://www.malacards.org/), DisGeNet database (https://www.disgenet.org/), and Comparative Toxicogenomics Database (http://ctdbase.org/, last update by June) obtain KOA-related targets.^[20,21]^ The target of the obtained compound was intersected with the KOA target, and the visual connection of “compound-drug-active ingredient-target protein-disease” was constructed.^[22]^ The raw data used in our investigation can be replicated and were taken from the relevant databases. Our tests’ outcomes are essentially unaffected by any disparities in the data, despite the fact that the databases are updated dynamically.

### 2.2. Construction of protein-protein interaction network

Protein-protein interactions underlie most BPs in living cells and are critical for understanding cell physiology in both normal and disease states. In this study, the protein-protein interaction (PPI) network analysis was performed on the obtained intersection gene sets using the string database (http://string-db.org/), and the species was restricted to “Homo sapiens” with a confidence value >0.4.^[23]^ The PPI network was constructed by Cytoscape software (version 3.9.1). In addition, using the plug-in CytoHubba algorithm of Cytoscape software, the targets with a maximum degree greater than or equal to half of the maximum degree were selected as core targets, and the top 10 targets in descending order of degree were selected as key targets.

### 2.3. GO, KEGG, and Reactome enrichment analysis

Import the obtained intersection targets into the HiPlot database (https://hiplot.com.cn/) and Reactome database (https://reactome.org/),^[24,25]^ and conduct pathways with humans as the research object enrichment analysis. Among them, Gene Ontology (GO) analysis includes 3 categories: biological process (BP), cellular component (CC), and molecular function, and according to the P. adjust. The top 20 enriched pathways of Reactome were selected according to the P. adjust. To select the top 20 annotated by KEGG, according to *P* value. The results are displayed in column charts, bubble charts, and network correlation diagrams.

### 2.4. Molecular docking analysis

The 3D structure of the proposed docking target in mol2 format from the Pubchem database, open the small ligand molecule with AutodockTools 1.5.6, perform hydrogenation, charging, detection of the ligand root, search and define rotatable bonds, and then save it as a pdbqt file. Download the core 3-dimensional structure of the target protein as a docking protein from the RCSB protein database (www.rcsb.org/). Add all hydrogen atoms in AutodockTools 1.5.6 to open, calculate the Gasteiger charge, combine nonpolar hydrogen, define it as an acceptor, and save it as a pdbqt file. Determine the coordinates and box size of the Vina molecular docking, set the exhaustiveness of the parameters to 15, and take the default values for other parameters. Autodockvina 1.1.2 was used for semiflexible docking, and the conformation with the best affinity was selected as the final docking conformation.

## 3. Result

### 3.1. Active ingredient and target protein analysis of SMYAD

**Figure 1.**
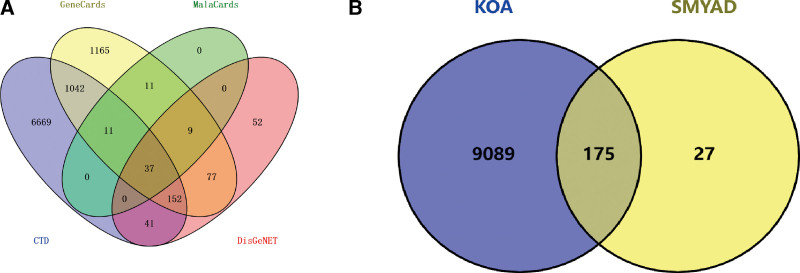
Target screening, (A) Venn diagram of disease target screening, (B) Venn diagram of SMYAD and KOA intersection targets. KOA = knee osteoarthritis, SMYAD = Simiao-Yongan Decoction.

**Figure 2.**
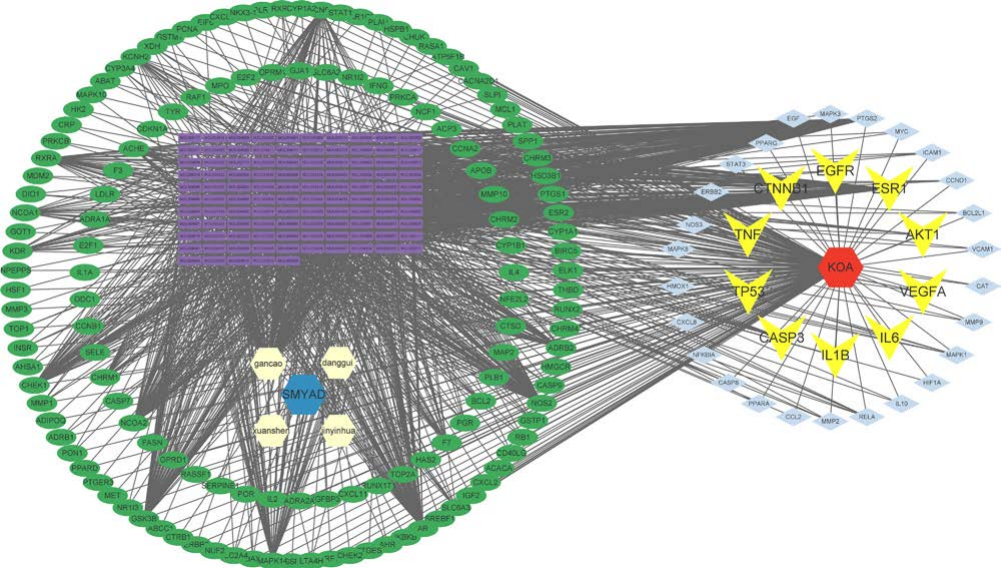
Network modulation diagram for SMYAD treatment of KOA. KOA = knee osteoarthritis, SMYAD = Simiao-Yongan Decoction.

Total of 126 active ingredients were retrieved through the TCMSP database, including 2 angelica, 23 honeysuckle, 9 Genuine Ginseng, and 92 licorice. After deleting repeated ingredients, a total of 120 chemical ingredients were obtained. There are a total of 2042 corresponding targets. The protein names were annotated according to the standard and the duplicate targets were deleted through the Uniprot database. A total of 202 targets were screened (Tables S1, Supplemental Digital Content, http://links.lww.com/MD/K40 and S2, http://links.lww.com/MD/K42). The keyword “Knee Osteoarthritis” was searched in the disease database, and the corresponding targets were obtained after screening, and the union was taken, and the KOA-related targets were obtained after deleting duplicate items (Table S3, Supplemental Digital Content, http://links.lww.com/MD/K43, Figure 1A). The targets corresponding to the active ingredients obtained through screening were intersected with KOA-related targets to obtain 175 intersection targets (Figure 1B). Use Cytoscape software to build a “compound-drug-active ingredient-target protein-disease” network (Figure 2). Using Cytoscape to calculate the weight of active ingredients, the top 8 active ingredients in degree value are quercetin, luteolin, kaempferol, beta-sitosterol, naringenin, 7-methoxy-2-methyl isoflavone, formononetin, licochalcone a. These may be the key active ingredients of SMYAD for the treatment of KOA (Table 1).

**Table 1:**
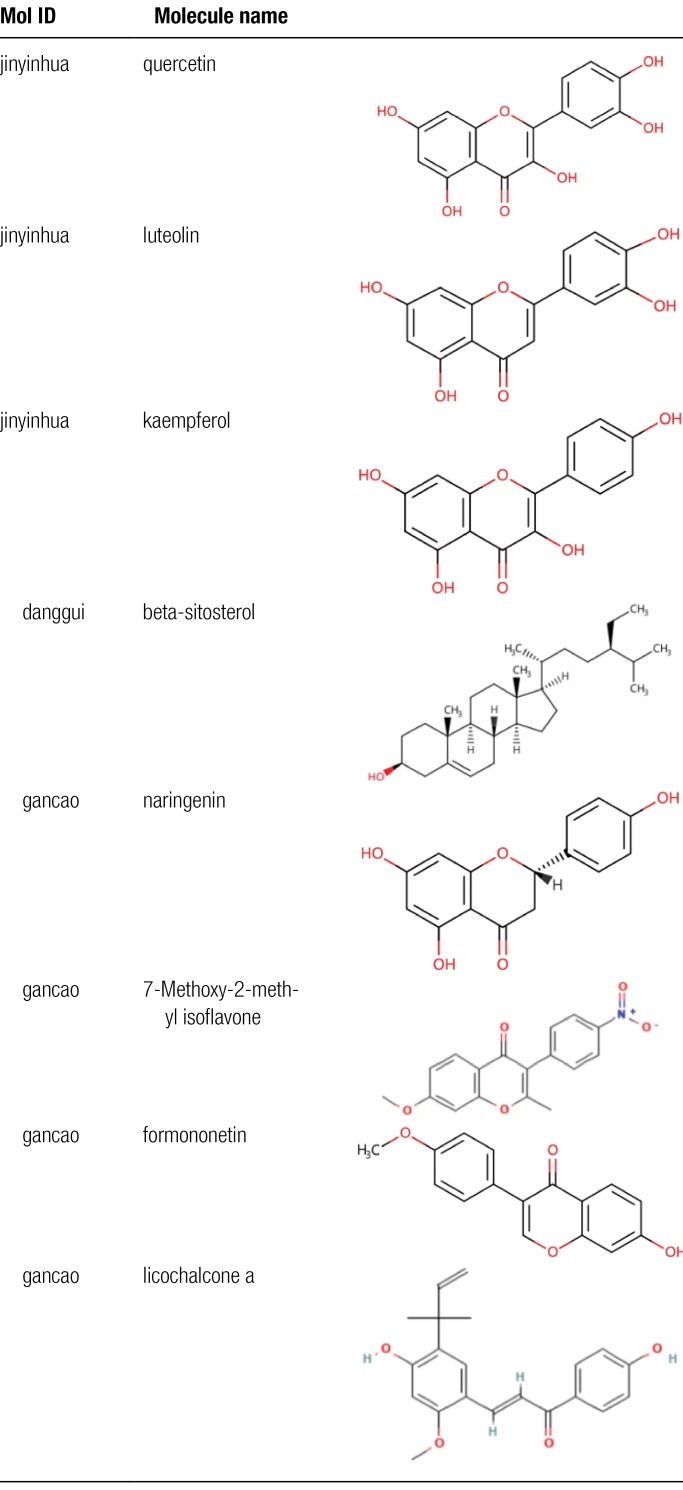
Screening of active ingredients of Simiao-Yongan Decoction from TCMSP database.

### 3.2. Construction of protein-protein interaction network

**Figure 3.**
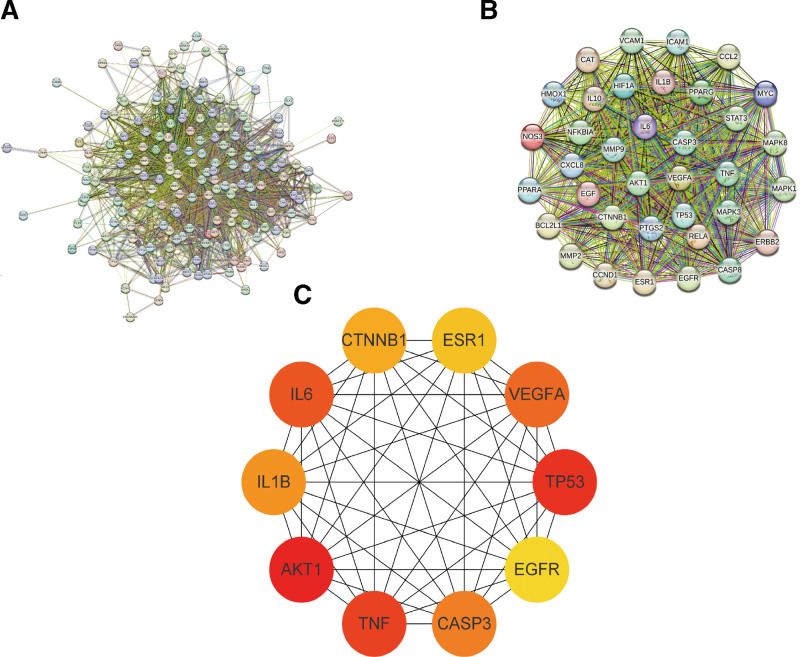
Core target and top 10 target data screening based on SMYAD and KOA intersection targets. KOA = knee osteoarthritis, SMYAD = Simiao-Yongan Decoction.

Import the intersection targets into the String database for PPI network. According to the CytoHubba algorithm, 36 core targets and the top 10 key targets of degree are obtained (Figure 3). The active ingredients corresponding to the core targets were intersected with the previously obtained 8 active ingredients that may be SMYAD for the treatment of KOA. The results showed that the intersection overlapped with the previous 8 active ingredients, so it was speculated that quercetin, luteolin, kaempferol, beta-sitosterol, naringenin, 7-methoxy-2-methyl isoflavone, formononetin, licochalcone a may be the key active ingredients of SMYAD in the treatment of KOA. And quercetin, luteolin, kaempferol, and beta-sitosterol contain the largest number of intersection targets, so they are considered to be the main effector substances.

### 3.3. Gene function and pathway enrichment results

**Figure 4.**
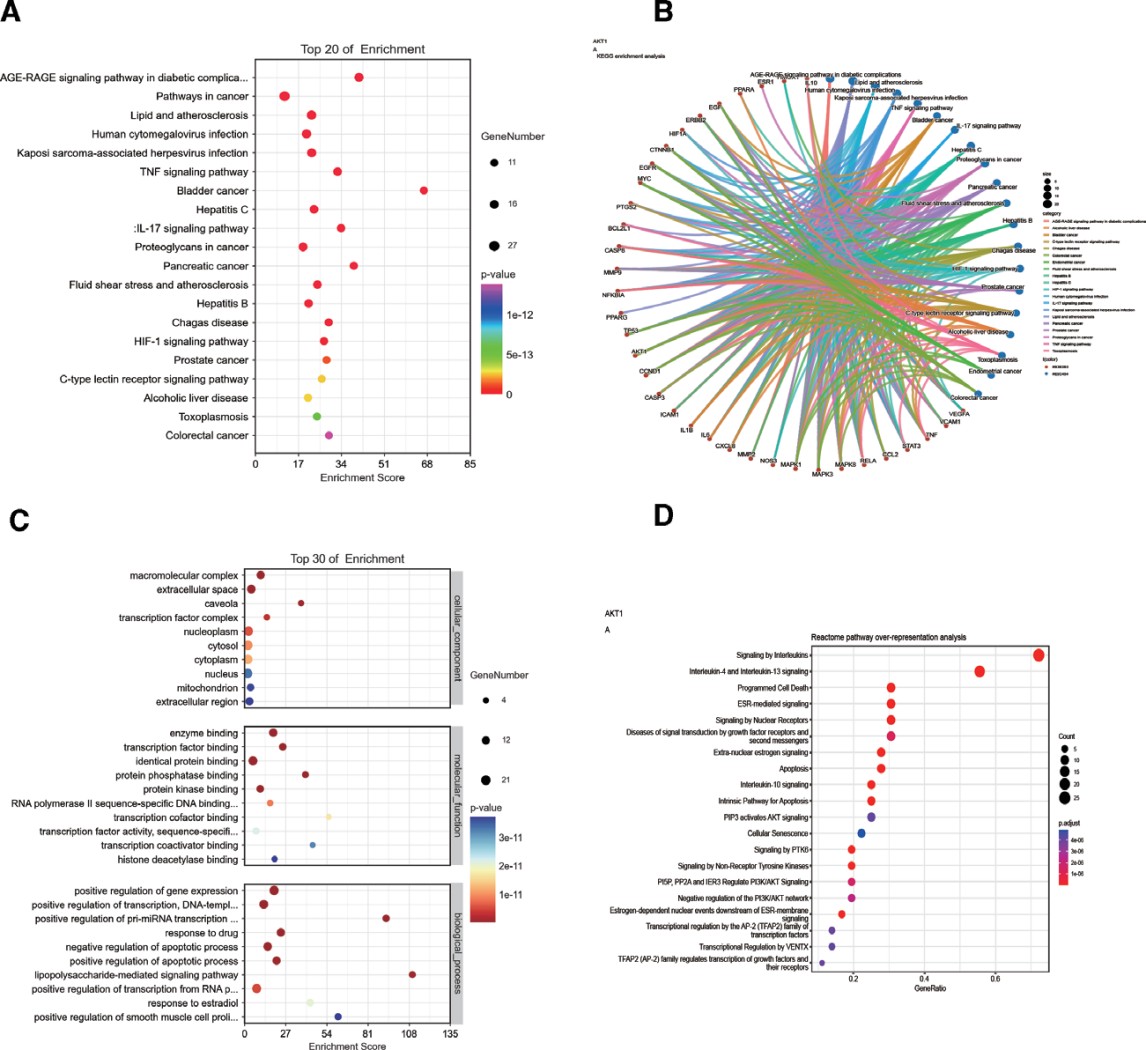
Results of the enrichment analysis graph. (A) KEGG enrichment analysis results graph, (B) signaling pathway and key target association maps based on KEGG enrichment analysis, (C) Barplot of the top 10 GO enrichment items, the GO enrichment items (BP, CC, MF) are arranged from up to low according to the adjusted P value, (D) bubble chart of Reactome enrichment analysis results. BP = biological processes, KEGG = Kyoto Encyclopedia of genes and genomes, CC = cell composition, GO = gene ontology, MF = molecular function.

**Figure 5.**
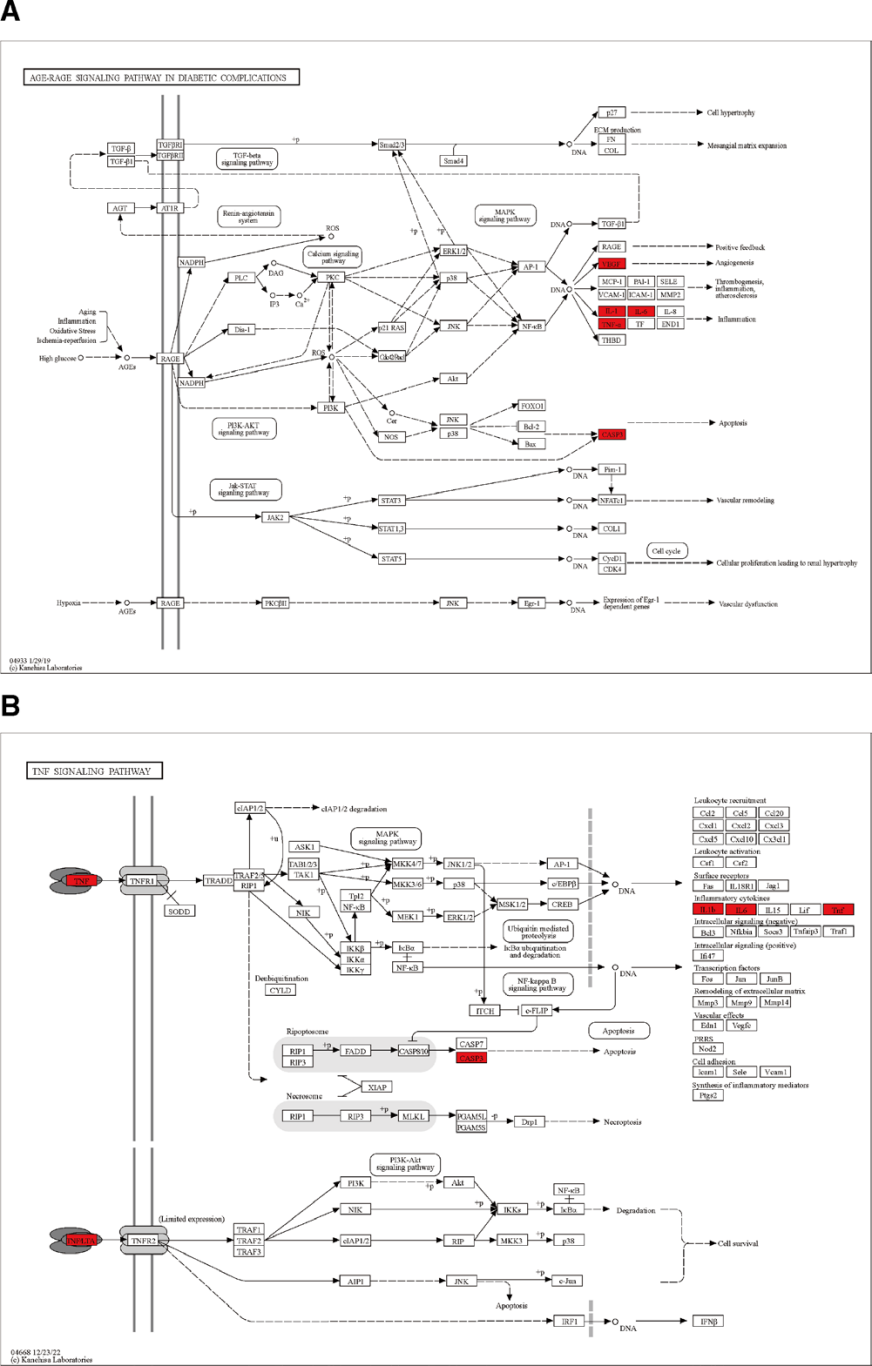
KEGG signaling pathway diagram, (A) AGE-RAGE signaling pathway in diabetic complications, (B) TNF signaling pathway. KEGG = Kyoto encyclopedia of genes and genomes, TNF = tumor necrosis factor.

**Figure 6.**
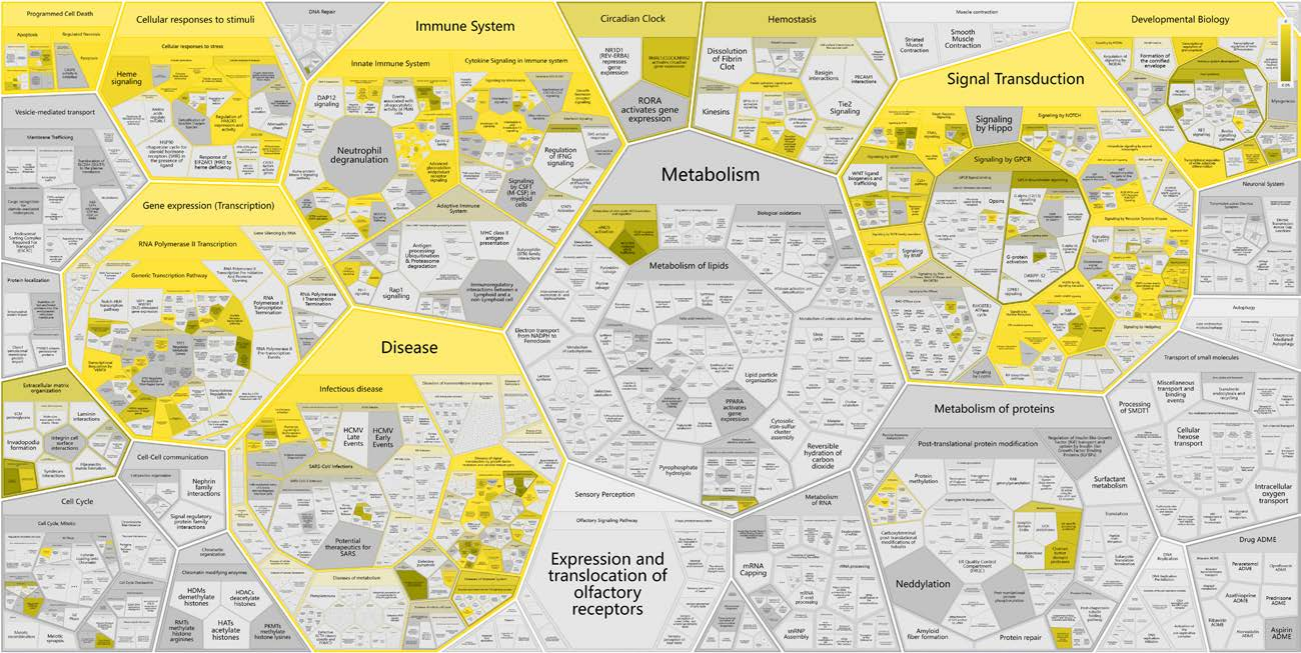
Reactome analysis. The diagrams show the human systems and locations of cellular responses involved in the key targets’ actions, and so on. The 36 key targets focus on the immune system, among these, the mechanisms involved in the immune system make it a priority for us to understand and study in depth.

Through the enrichment analysis of 36 core targets, a total of 536 GO annotations were obtained. According to the value of P. adjust, the top 10 BP, CC, and molecular function of GO annotations were screened out (Table S4, Supplemental Digital Content, http://links.lww.com/MD/K44). Among them, BP mainly involves positive regulation of gene expression, positive regulation of transcription, DNA-templated, positive regulation of pri-miRNA transcription from RNA polymerase II promoter, response to drug, negative regulation of apoptotic process, positive regulation of apoptotic process, lipopolysaccharide-mediated signaling pathway, positive regulation of transcription from RNA polymerase II promoter, response to estradiol, positive regulation of smooth muscle cell proliferation. CC mainly involves macromolecular complex, extracellular space, Caveola, transcription factor complex, nucleoplasm, cytosol, cytoplasm, nucleus, mitochondrion, extracellular region. MM mainly includes enzyme binding, transcription factor binding, identical protein binding, protein phosphatase binding, protein kinase binding, RNA polymerase II sequence-specific DNA binding transcription factor binding, transcription cofactor binding, transcription factor activity, sequence-specific DNA binding, transcript coactivator binding, histone deacetylase binding (Fig 4C, Table 2). One hundred forty-six pathways were enriched by KEGG, and the top 20 annotated by KEGG were screened out according to the value of *P* value. Its main pathway involves AGE-RAGE signaling pathway in diabetic complications, pathways in cancer, lipid and atherosclerosis, HIF-1 signaling pathway, TNF signaling pathway, IL-17 signaling pathway (Fig. 4A,B; Table 3). Reactome was enriched to 228 pathways. According to the value of P. adjust, the top 20 pathways were screened out, mainly involving interleukin-4 and interleukin-13 signaling, signaling by interleukins, cytokine signaling in immune system, immune system, interleukin-10 signaling, apoptosis (Figure 4D, Table 4). According to the results of comprehensive enrichment analysis, the main effect pathways of SMYAD in treating KOA are AGE-RAGE signaling pathway in diabetic complications and TNF signaling pathway. We downloaded the relevant pathway diagrams from the KEGG database for further analysis and found that IL6, TNF, CASP3, and IL1B are the key regulatory targets of the 2 pathways, and they highly overlap with the top 10 key proteins we screened (Figure 5). In addition, the 2 signaling pathways ultimately affect the expression of apoptotic signals, and these results are consistent with the enrichment analysis results of GO and Reactome (Figure 6). Therefore, we believe that based on the key proteins IL6, TNF, CASP3, and IL1B, the regulation of AGE-RAGE signaling pathway in diabetic complications and TNF signaling pathway to affect the occurrence of apoptosis is the key to the treatment of KOA by SMYAD.

**Table 2:** The top 10 gene ontology enrichment items.

ID	Term	Category	p.value
GO:0010628	Positive regulation of gene expression	biological_process	1.40E-19
GO:0045893	Positive regulation of transcription, DNA-templated	biological_process	2.65E-14
GO:1902895	Positive regulation of pri-miRNA transcription from RNA polymerase II promoter	biological_process	3.47E-14
GO:0042493	Response to drug	biological_process	7.17E-14
GO:0043066	Negative regulation of apoptotic process	biological_process	1.56E-13
GO:0043065	Positive regulation of apoptotic process	biological_process	3.16E-13
GO:0031663	Lipopolysaccharide-mediated signaling pathway	biological_process	4.96E-13
GO:0045944	Positive regulation of transcription from RNA polymerase II promoter	biological_process	4.81E-12
GO:0032355	Response to estradiol	biological_process	2.03E-11
GO:0048661	Positive regulation of smooth muscle cell proliferation	biological_process	3.72E-11
GO:0032991	Macromolecular complex	cellular_component	9.33E-10
GO:0005615	Extracellular space	cellular_component	1.43E-06
GO:0005901	Caveola	cellular_component	8.68E-06
GO:0005667	Transcription factor complex	cellular_component	4.47E-05
GO:0005654	Nucleoplasm	cellular_component	8.43E-05
GO:0005829	Cytosol	cellular_component	1.47E-04
GO:0005737	Cytoplasm	cellular_component	1.79E-04
GO:0005634	Nucleus	cellular_component	5.09E-04
GO:0005739	Mitochondrion	cellular_component	5.54E-04
GO:0005576	Extracellular region	cellular_component	5.65E-04
GO:0019899	Enzyme binding	molecular_function	9.82E-14
GO:0008134	Transcription factor binding	molecular_function	1.14E-10
GO:0042802	Identical protein binding	molecular_function	1.74E-09
GO:0019903	Protein phosphatase binding	molecular_function	1.63E-08
GO:0019901	Protein kinase binding	molecular_function	2.71E-07
GO:0061629	RNA polymerase II sequence-specific DNA binding transcription factor binding	molecular_function	2.35E-05
GO:0001221	Transcription cofactor binding	molecular_function	4.69E-05
GO:0003700	Transcription factor activity, sequence-specific DNA binding	molecular_function	5.95E-05
GO:0001223	Transcription coactivator binding	molecular_function	8.91E-05
GO:0042826	Histone deacetylase binding	molecular_function	1.03E-04

**Table 3:** The top 20 KEGG enrichment items.

ID	Term	*P*
hsa04933	AGE-RAGE signaling pathway in diabetic complications	2.62E-24
hsa05200	Pathways in cancer	2.71E-24
hsa05417	Lipid and atherosclerosis	2.17E-23
hsa05163	Human cytomegalovirus infection	2.66E-21
hsa05167	Kaposi sarcoma-associated herpesvirus infection	7.55E-21
hsa04668	TNF signaling pathway	1.03E-19
hsa05219	Bladder cancer	4.27E-18
hsa05160	Hepatitis C	1.96E-17
hsa04657	IL-17 signaling pathway	2.94E-17
hsa05205	Proteoglycans in cancer	3.34E-17
hsa05212	Pancreatic cancer	1.12E-16
hsa05418	Fluid shear stress and atherosclerosis	1.38E-16
hsa05161	Hepatitis B	1.23E-15
hsa05142	Chagas disease	4.64E-15
hsa04066	HIF-1 signaling pathway	1.05E-14
hsa05215	Prostate cancer	1.17E-13
hsa04625	C-type lectin receptor signaling pathway	2.56E-13
hsa04936	Alcoholic liver disease	2.68E-13
hsa05145	Toxoplasmosis	5.90E-13
hsa05210	Colorectal cancer	1.39E-12

**Table 4:** The top 10 Reactome enrichment items.

ID	Pathway	*P*
R-HSA-6785807	Interleukin-4 and interleukin-13 signaling	5.21E-30
R-HSA-449147	Signaling by interleukins	8.32E-29
R-HSA-1280215	Cytokine signaling in immune system	6.78E-24
R-HSA-168256	Immune system	2.26E-16
R-HSA-6783783	Interleukin-10 signaling	1.33E-12
R-HSA-9009391	Extranuclear estrogen signaling	1.59E-12
R-HSA-109606	Intrinsic pathway for apoptosis	5.07E-12
R-HSA-162582	Signal transduction	3.46E-11
R-HSA-5357801	Programmed cell death	7.57E-10
R-HSA-8939211	ESR-mediated signaling	1.18E-09
R-HSA-9006934	Signaling by receptor tyrosine kinases	3.35E-09
R-HSA-109581	Apoptosis	3.52E-09
R-HSA-1643685	Disease	3.96E-09
R-HSA-2262752	Cellular responses to stress	5.36E-09
R-HSA-8953897	Cellular responses to stimuli	6.82E-09
R-HSA-9634638	Estrogen-dependent nuclear events downstream of ESR-membrane signaling	1.01E-08
R-HSA-8848021	Signaling by PTK6	1.58E-08
R-HSA-9006927	Signaling by nonreceptor tyrosine kinases	1.58E-08
R-HSA-9006931	Signaling by nuclear receptors	2.00E-08
R-HSA-168142	Toll-like receptor 10 (TLR10) cascade	5.46E-07

### 3.4. Molecular docking

**Figure 7.**
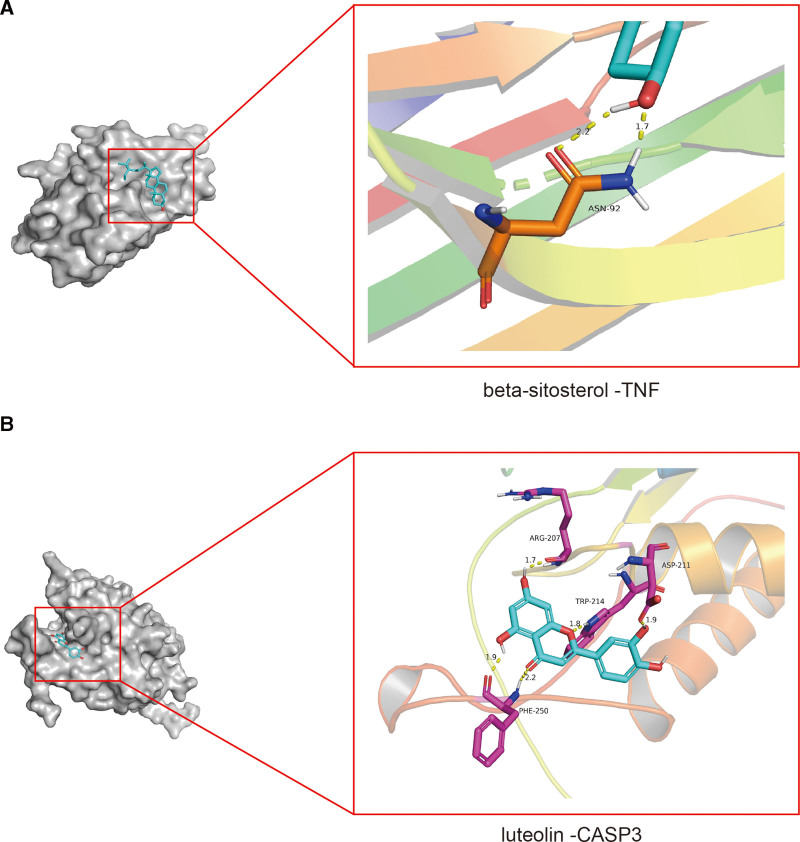
Molecular docking results of “bioactive compound-hub gene.”

According to the results of enrichment analysis, we found that TNF, IL6, IL1B, and CAPSE3, the key proteins of SMYAD in the treatment of KOA, play an important role. Therefore, we carried out molecular docking of these 4 hub genes and important active compounds quercetin, luteolin, kaempferol, beta-sitosterol. Small molecule ligands can spontaneously bind to protein receptors when the binding energy is <0 kJ mol. If the binding energy is <−5.0 kJ mol or lower, it indicates that the 2 have better binding ability. Through the docking simulation, 16 docking results were generated (Table 5). Their binding energies are all <0 kJ mol, which means they all bind well. Finally, Pymol software was used to visualize the complexes of 4 groups of proteins and compounds with good docking effect (Figure 7).

**Table 5:** The binding energy of molecular docking (kJ mol).

Active chemicals hub genes	TNF	IL6	IL1B	CAPSE3
Quercetin	−6.40	−5.47	−6.06	−7.32
Luteolin	−6.28	−6.02	−6.88	−8.04
Kaempferol	−6.58	−6.05	−6.07	−6.81
Beta-sitosterol	−8.14	−6.11	−5.74	−7.86

## 4. Discussion

KOA is a kind of degenerative arthritis that mainly occurs in the elderly. It can be caused by a variety of causes, including articular cartilage damage, subchondral bone tissue, joint edge synovium, and joint tissue changes.^[26]^ Clinically, in the face of patients with knee arthritis in the early and middle stages, nonsurgical treatment is often used to improve knee joint disorders, relieve pain, promote joint function recovery, and reduce articular cartilage damage.^[27]^ Commonly used methods include active muscle strength training, activity adjustment, and changing activity patterns, and so on, and most patients can obtain relatively ideal therapeutic effects.^[28]^ For patients with advanced knee arthritis because the articular cartilage is severely damaged and the bone is exposed and necrotic, extensive osteophytes, and subchondral cysts are prone to occur. Although there is no specific treatment at present, joint cavity washing can effectively relieve the pain, but the curative effect is not obvious.^[29]^ In recent years, many experiments and clinical observations have confirmed that TCM has a good curative effect on KOA,^[30]^ among which SMYAD has a good therapeutic effect on KOA. However, its active ingredients and mechanism are still unclear. Therefore, this study intends to discover the potential mechanism of SMYAD in the treatment of KOA through network pharmacology, and provide theoretical support for the application of TCM.

One hundred twenty active ingredients of SMYAD were screened out from the TCMSP database. The top 8 active ingredients ranked according to degree value are quercetin, luteolin, kaempferol, beta-sitosterol, naringenin, 7-methoxy-2-methyl isoflavone, formononetin, licochalcone a. Therefore, it is speculated that these may be the key active ingredients of SMYAD in the treatment of KOA. Quercetin is a polyhydroxy flavonoid compound, which has biological activities such as antivirus, antitumor, and liver protection.^[31]^ Related studies have found that quercetin can up-regulate SOD and TIMP-1, down-regulate MMP-13, and improve the degeneration of KOA.^[32]^ Luteolin is a natural flavonoid found in many plants. It has a variety of pharmacological activities, such as antiinflammatory, antiallergic, and uric acid-lowering.^[33]^ Studies have shown that luteolin can regulate the gene expression, secretion, and activity of MMP-3 by acting directly on articular chondrocytes, and play a potential chondroprotective role.^[34]^ Kaempferol is also a kind of natural flavonoids, mainly found in tea, broccoli, apple, and other plants. A large number of clinical studies have shown that kaempferol and some kaempferol glycosides have a wide range of pharmacological activities, including antioxidation, antiinflammation, antidiabetes, antiosteoporosis and other effects.^[35]^ Studies have found that kaempferol potentially improves the efficiency of arthritis cell therapy.^[36]^ The above studies are consistent with the results of this study, suggesting that quercetin, luteolin, kaempferol, and other active ingredients may play a key role in the treatment of OP by SMYAD. According to Degree analysis of PPI network, the results showed that 10 core targets AKT1, TP53, TNF, IL6, VEGF-A, CASP3, IL1B, CTNNB1, ESR1, EGFR may play a key role in the treatment of KOA by SMYAD. VEGF is a well-known angiogenic factor. Related studies have shown that the expression of VEGF-A is a special feature of arthritis, and found that VEGF-A and VEGFR2 are related to the vascular invasion of cartilage.^[37]^ Elevated VEGF-A levels are associated with the progression of OA. It is involved in the specific pathological processes of OA, including cartilage degeneration, osteophyte formation, subchondral bone cysts and sclerosis, synovitis and pain. Inhibition of VEGF-A signaling can reduce the progression of OA.^[38]^ The results of GO functional enrichment analysis suggested that SMYAD treatment of KOA mainly involves positive regulation of gene expression, negative regulation of apoptosis process, and positive regulation of apoptosis process. KEGG signaling pathway mainly involves AGE-RAGE signaling pathway in diabetic complications, TNF signaling pathway, HIF-1 signaling pathway, IL-17 signaling pathway. The pathway of Recactome mainly involves interleukin-4 and interleukin-13 signaling, cytokine signaling in immune system, immune system, apoptosis. The biological activity of AGEs is considered to be mediated by age-specific receptors (RAGE), and the activation of RAGE is involved in key signaling pathways related to KOA.^[39]^ On the one hand, AGEs induce inflammation by activating NF-κB and MAPK in various cell types including chondrocytes. On the other hand, AGEs may increase the levels of PGE and NO through the MAPK pathway to enhance the inflammatory response of OA chondrocytes. In addition, AGEs stimulated endoplasmic reticulum stress in human chondrocytes through RAGE, leading to phosphorylation of eIF2α and p38 MAP kinases and activation of NF-κB, which in turn increased COX-2 expression and PGE2 production.^[40]^ IL-17 is one of many inflammatory cytokines, mainly produced by T cells. The cells affected by IL-17 in joints are mainly chondrocytes. Imaging studies have shown that images of OA lesions are positively correlated with elevated levels of IL-17 in serum and synovial fluid. In addition, IL-17 can induce chondrocytes and synovial fibroblasts to release chemokines and induce chondrocytes to synthesize IL-1, thereby promoting the pathological progression of OA.^[41]^ Hypoxia-inducible factor (HIF) is a transcription factor and a key regulator of cellular responses to hypoxia.^[42]^ HIF-1 is an important factor in maintaining chondrocyte homeostasis and allowing cell differentiation. HIF-1 is a heterodimer composed of 2 subunits: HIF-1α and HIF-1. A study showed that HIF-1α plays an important role in the prevention of OA. HIF-1α downregulates MMP-13 by inhibiting β-catenin transcriptional activity in chondrocytes, thereby alleviating OA development. Lack of HIF-1α exacerbates cartilage catabolism. In addition, HIF-1 is also involved in the process of KOA synovial fibrosis.^[43]^ Molecular docking showed that the important active compounds quercetin, luteolin, kaempferol, and beta-sitosterol could fully combine with key proteins TNF, IL6, IL1B, and CAPSE3. This may be the key molecular mechanism of SMYAD in treating KOA.

Even while our research has clearly shown the beneficial efficacy and mechanism of SMYAD in the treatment of KOA, our medication is safer for clinical use when compared to the side effects of conventional NSAIDs, such as peptic ulcers. Nonetheless, there are still certain issues that require improvement. First of all, SMYAD is a complex formula with a wide range of effects from each of its constituent parts. Therefore, a review of the drug’s plasmapheresis analysis is necessary to further elucidate the effects of each component. The few active chemicals we detected may not be sufficient. Second, until date, we have only verified theoretical partial molecular docking in a thorough and scientific manner. We plan to enhance the associated experiments in the future.

## 5. Conclusion

In summary, this study preliminarily verified and predicted the molecular mechanism of SMYAD in the treatment of KOA, and found that the active ingredients and mechanism of SMYAD in the treatment of KOA present the characteristics of multi-component, multi-target, and multi - pathway.

## Acknowledgments

We would like to thank Sichuan Vocational College of Health and Rehabilitation for helpful discussions on topics relevant to this work.

## Author contributions

**Conceptualization:** Lan Chen.

**Project administration:** Junwei Wang.

**Software:** Haixu Chen.

**Validation:** Xiangyu Pan.

**Writing – original draft:** Ying Wang.

## Supplementary Material

## References

[1] Sharma L. Osteoarthritis of the Knee. N Engl J Med. 2021;384:51–9. 33406330 10.1056/NEJMcp1903768

[2] Mahmoudian A Lohmander LS Mobasheri A. Early-stage symptomatic osteoarthritis of the knee - time for action. Nat Rev Rheumatol. 2021;17:621–32. 34465902 10.1038/s41584-021-00673-4

[3] Jang S Lee K Ju JH. Recent updates of diagnosis, pathophysiology, and treatment on osteoarthritis of the knee. Int J Mol Sci . 2021;22:2619. 33807695 10.3390/ijms22052619 PMC7961389

[4] Quinn RH Murray JN Pezold R. Surgical management of osteoarthritis of the knee. J Am Acad Orthop Surg. 2018;26:e191–3. 29688919 10.5435/JAAOS-D-17-00424

[5] Jamshidi A Pelletier JP Martel-Pelletier J. Machine-learning-based patient-specific prediction models for knee osteoarthritis. Nat Rev Rheumatol. 2019;15:49–60. 30523334 10.1038/s41584-018-0130-5

[6] Hussain SM Neilly DW Baliga S. Knee osteoarthritis: a review of management options. Scott Med J. 2016;61:7–16. 27330013 10.1177/0036933015619588

[7] Belluzzi E Olivotto E Toso G. Conditioned media from human osteoarthritic synovium induces inflammation in a synoviocyte cell line. Connect Tissue Res. 2019;60:136–45. 29695173 10.1080/03008207.2018.1470167

[8] Biz C Maso G Gambato M. Challenging surgical treatment of displaced articular tibial plateau fractures: do early knee radiographic features have a predictive value of the mid-term clinical functional outcomes? Orthop Surg 2019;11:1149–62. 31755217 10.1111/os.12577 PMC6904635

[9] Lee R Kean WF. Obesity and knee osteoarthritis. Inflammopharmacology 2012;20:53–8. 22237485 10.1007/s10787-011-0118-0

[10] Allaeys C Arnout N Van Onsem S. Conservative treatment of knee osteoarthritis. Acta Orthop Belg. 2020;86:412–21. 33581025

[11] Jeon OH Kim C Laberge RM. Local clearance of senescent cells attenuates the development of post-traumatic osteoarthritis and creates a pro-regenerative environment. Nat Med. 2017;23:775–81. 28436958 10.1038/nm.4324 PMC5785239

[12] Katz JN Arant KR Loeser RF. Diagnosis and treatment of hip and knee osteoarthritis: a review. JAMA. 2021;325:568–78. 33560326 10.1001/jama.2020.22171 PMC8225295

[13] Jones IA Togashi R Wilson ML. Intra-articular treatment options for knee osteoarthritis. Nat Rev Rheumatol. 2019;15:77–90. 30498258 10.1038/s41584-018-0123-4 PMC6390843

[14] Zappavigna S Cossu AM Grimaldi A. Anti-inflammatory drugs as anticancer agents. Int J Mol Sci . 2020;21:2605. 32283655 10.3390/ijms21072605 PMC7177823

[15] Jie SS Sun HJ Liu JX. Simiao Yong’an decoction ameliorates murine collagen-induced arthritis by modulating neutrophil activities: An in vitro and in vivo study. J Ethnopharmacol. 2023;305:116119. 36596398 10.1016/j.jep.2022.116119

[16] Du A Xie Y Ouyang H. Si-Miao-Yong-an decoction for diabetic retinopathy: a combined network pharmacological and in vivo approach. Front Pharmacol. 2021;12:763163. 34899317 10.3389/fphar.2021.763163 PMC8661904

[17] Li X Wei S Niu S. Network pharmacology prediction and molecular docking-based strategy to explore the potential mechanism of Huanglian Jiedu Decoction against sepsis. Comput Biol Med. 2022;144:105389. 35303581 10.1016/j.compbiomed.2022.105389

[18] Dong Y Zhao Q Wang Y. Network pharmacology-based investigation of potential targets of astragalus membranaceous-angelica sinensis compound acting on diabetic nephropathy. Sci Rep. 2021;11:19496. 34593896 10.1038/s41598-021-98925-6 PMC8484574

[19] Fan JH Xu MM Zhou LM. Integrating network pharmacology deciphers the action mechanism of Zuojin capsule in suppressing colorectal cancer. Phytomedicine. 2022;96:153881. 34942456 10.1016/j.phymed.2021.153881

[20] Weijiao Y Fuchun L Mengjie C. Immune infiltration and a ferroptosis-associated gene signature for predicting the prognosis of patients with endometrial cancer. Aging (Albany NY) 2021;13:16713–32. 34170849 10.18632/aging.203190 PMC8266342

[21] Davis AP Grondin CJ Johnson RJ. Comparative Toxicogenomics Database (CTD): update 2021. Nucleic Acids Res. 2021;49:D1138–43. 33068428 10.1093/nar/gkaa891 PMC7779006

[22] Otasek D Morris JH Bouças J. Cytoscape Automation: empowering workflow-based network analysis. Genome Biol. 2019;20:185. 31477170 10.1186/s13059-019-1758-4 PMC6717989

[23] Chen YC Chen YH Wright JD. PPI-Hotspot(DB): Database of protein-protein interaction hot spots. J Chem Inf Model. 2022;62:1052–60. 35147037 10.1021/acs.jcim.2c00025

[24] Griss J Viteri G Sidiropoulos K. ReactomeGSA - efficient multi-omics comparative pathway analysis. Mol Cell Proteomics. 2020;19:2115–25. 32907876 10.1074/mcp.TIR120.002155 PMC7710148

[25] Fabregat A Korninger F Viteri G. Reactome graph database: Efficient access to complex pathway data. PLoS Comput Biol. 2018;14:e1005968. 29377902 10.1371/journal.pcbi.1005968 PMC5805351

[26] Michael JW Schlüter-Brust KU Eysel P. The epidemiology, etiology, diagnosis, and treatment of osteoarthritis of the knee. Dtsch Arztebl Int 2010;107:152–62. 20305774 10.3238/arztebl.2010.0152 PMC2841860

[27] Kan HS Chan PK Chiu KY. Non-surgical treatment of knee osteoarthritis. Hong Kong Med J. 2019;25:127–33. 30919810 10.12809/hkmj187600

[28] Perlman A Fogerite SG Glass O. Efficacy and safety of massage for osteoarthritis of the knee: a Randomized Clinical Trial. J Gen Intern Med. 2019;34:379–86. 30543021 10.1007/s11606-018-4763-5 PMC6420526

[29] Hall M van der Esch M Hinman RS. How does hip osteoarthritis differ from knee osteoarthritis? Osteoarthritis Cartilage. 2022;30:32–41. 34600121 10.1016/j.joca.2021.09.010

[30] Liang Y Xu Y Zhu Y. Efficacy and safety of chinese herbal medicine for knee osteoarthritis: Systematic Review and Meta-analysis of Randomized Controlled Trials. Phytomedicine. 2022;100:154029. 35316726 10.1016/j.phymed.2022.154029

[31] Cui Z Zhao X Amevor FK. Therapeutic application of quercetin in aging-related diseases: SIRT1 as a potential mechanism. Front Immunol. 2022;13:943321. 35935939 10.3389/fimmu.2022.943321 PMC9355713

[32] Wei B Zhang Y Tang L. Protective effects of quercetin against inflammation and oxidative stress in a rabbit model of knee osteoarthritis. Drug Dev Res. 2019;80:360–7. 30609097 10.1002/ddr.21510

[33] Imran M Rauf A Abu-Izneid T. Luteolin, a flavonoid, as an anticancer agent: A review. Biomed Pharmacother. 2019;112:108612. 30798142 10.1016/j.biopha.2019.108612

[34] Kang BJ Ryu J Lee CJ. Luteolin inhibits the activity, secretion and gene expression of MMP-3 in cultured articular chondrocytes and production of MMP-3 in the rat knee. Biomol Ther (Seoul) 2014;22:239–45. 25009705 10.4062/biomolther.2014.020 PMC4060084

[35] Dabeek WM Marra MV. Dietary quercetin and kaempferol: bioavailability and potential cardiovascular-related bioactivity in humans. Nutrients 2019;11:2288. 31557798 10.3390/nu11102288 PMC6835347

[36] Estakhri F Panjehshahin MR Tanideh N. The effect of kaempferol and apigenin on allogenic synovial membrane-derived stem cells therapy in knee osteoarthritic male rats. Knee 2020;27:817–32. 32336589 10.1016/j.knee.2020.03.005

[37] Qian JJ Xu Q Xu WM. Expression of VEGF-A signaling pathway in cartilage of ACLT-induced Osteoarthritis Mouse Model. J Orthop Surg Res 2021;16:379. 34127028 10.1186/s13018-021-02528-w PMC8201729

[38] Zhu Y Zhong W Peng J. Study on the mechanism of baimai ointment in the treatment of osteoarthritis based on network pharmacology and molecular docking with experimental verification. Front Genet. 2021;12:750681. 34868222 10.3389/fgene.2021.750681 PMC8635803

[39] Steenvoorden MM Huizinga TW Verzijl N. Activation of receptor for advanced glycation end products in osteoarthritis leads to increased stimulation of chondrocytes and synoviocytes. Arthritis Rheum. 2006;54:253–63. 16385542 10.1002/art.21523

[40] Nah SS Choi IY Lee CK. Effects of advanced glycation end products on the expression of COX-2, PGE2 and NO in human osteoarthritic chondrocytes. Rheumatology (Oxford) 2008;47:425–31. 18285354 10.1093/rheumatology/kem376

[41] Roman-Blas JA Jimenez SA. NF-kappaB as a potential therapeutic target in osteoarthritis and rheumatoid arthritis. Osteoarthritis Cartilage. 2006;14:839–48. 16730463 10.1016/j.joca.2006.04.008

[42] Fernández-Torres J Zamudio-Cuevas Y Martínez-Nava GA. Hypoxia-Inducible Factors (HIFs) in the articular cartilage: a systematic review. Eur Rev Med Pharmacol Sci. 2017;21:2800–10. 28682438

[43] Bouaziz W Sigaux J Modrowski D. Interaction of HIF1α and β-catenin inhibits matrix metalloproteinase 13 expression and prevents cartilage damage in mice. Proc Natl Acad Sci U S A. 2016;113:5453–8. 27122313 10.1073/pnas.1514854113 PMC4868441

